# Study of *in vitro* and *in vivo* effects of 1,6-Bis[4-(4-amino-3-hydroxyphenoxy)phenyl]diamantane (DPD), a novel cytostatic and differentiation inducing agent, on human colon cancer cells

**DOI:** 10.1038/sj.bjc.6601337

**Published:** 2003-11-11

**Authors:** J J Wang, Y F Chang, Y T Chern, C W Chi

**Affiliations:** 1 National Taipei College of Nursing, 365 Ming Te Road, Taipei 11219, Taiwan; 2 Department of Medical Research and Education, Taipei Veterans General Hospital, Taiwan; 3 Department of Chemical Engineering, National Taiwan University of Science and Technology, Taiwan; 4 Institute of Pharmacology, School of Medicine, National Yang-Ming University, Taipei, Taiwan

**Keywords:** Colo 205, HT-29, HCT-15, brush border, cyclin D, cytostatic

## Abstract

A diamantane derivative 1,6-Bis [4-(4-amino-3-hydroxyphenoxy) phenyl] diamantane (DPD) was found to inhibit the growth of several cancer cell lines in the National Cancer Institute (NCI) Anticancer Drug Screen system. In this study, we examined the *in vitro* and *in vivo* effects of DPD on human colon cancer cells. DPD exerted growth inhibitory activities *in vitro* against three human colon cancer cell lines (Colo 205, HT-29, and HCT-15). DPD-treated cells were arrested at G_0_/G_1_ as analysed by flow cytometric analysis. The expression of cyclin D was decreased in DPD-treated cells. The differentiation markers of carcinoembryonic antigen and fibronectin were significantly increased in colon cancer cells after treatment with DPD. The epithelium-like brush borders on HT-29 cell surface were also demonstrated at 1 week after withdrawal from DPD treatment. The DPD-induced cell growth inhibition and differentiation were irreversible after removal of DPD. The *in vivo* effect of tumour growth suppression by DPD was also observed in mouse xenografts. No acute toxicity was observed after an intraperitoneal challenge of DPD in BALB/c-nude mice weekly. These results suggest that DPD appears to be a new potentially less toxic modality of cancer therapy.

Adamantane derivatives possess several attractive pharmacological activities, such as antibacterial, antifungal, antiviral, and anticancer effects (Aigami *et al*, 1975; Wang *et al*, 1997, 1998). Our previous study has found that *N*-1-adamantylcitraconimide, *N*-1-adamantylmaleimide (AMI) and *N*-1-diamantylmaleimide (DMI) exhibited modest growth-inhibitory activity against four cancer cell lines (Colo 205, HepG2, SK-BR-3, and Molt-4), and AMI and DMI exhibited antimicrobial activity against *Staphylococcus aureus* and *Trichophyton mentagrophytes* (Wang *et al*, 1997). We also found that AMI was effective in inhibiting the growth of human gastric cancer cells both *in vitro* and *in vivo* and induced apoptosis *in vitro* (Wang *et al*, 1998). The AMI derivative dimethyladamantylmaleimide (DMAMI) induces apoptosis and inhibits the growth of the human colon cancer Colo205 in SCID mice (Wang *et al*, 2002). AMI-induced morphological changes of the cell membrane may lead to apoptosis of SC-M1 cells (Wang *et al*, 1998). We suggest that the cell membrane may be the target of adamantane. Adamantane and diamantane are closely analogous polycyclic alkane with the structure of three and six fused cyclohexane rings, respectively. Although diamantane derivatives have been investigated by chemists for many years, only a few studies of biological activity on diamantane derivatives are reported (Chen *et al*, 1994). In a recent study, we have characterised the anticancer activities of diamantane derivatives using the 60 human cancer cell lines in NCI Anticancer Drug Screen, and evaluated the structure–activity relationship (unpublished data). 1,6-Bis[4-(4-amino-3-hydroxyphenoxy) phenyl] diamantane (DPD) exhibited marked anticancer activities on the subpanel of 60 human cancer cell lines, especially the Colo 205 (unpublished data).


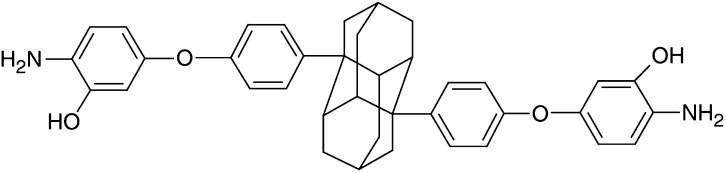


For example, very strong anticancer effects of DPD were observed against one leukaemia (HL-60), one non-small-cell lung cancer (HOP-92), one colon cancer (Colo 205), one ovarian cancer (OVCAR-8) and one breast cancer (T-47D) cell line with GI_50_ (LC_50_) of 0.50 (>100), 0.85 (22.3), 1.31 (6.24), 0.62 (>100) and 0.75 (>100) *μ*M, respectively (unpublished data).

Colon cancer is a major cause of mortality in the Western world (Giovannucci, 2002). Although chemotherapy and radiation therapy have been attempted in either adjuvant or palliative treatments, more effective adjuvant therapy is needed for colon cancer patients. Nearly half of all patients with colon cancer still die of metastatic disease after curative surgery (NIH Consensus Conference, 1990). Therefore, developing new therapeutic drugs for colon cancer is a worthwhile task.

Disordered proliferation is one of the characteristics of most of malignant tumours. Multiple genetic alterations in tumour cells affect the regulation of the cell cycle machinery (Hunter and Pines, 1994). Therefore, restoring normal cell cycle control in tumour cells by using small molecule inhibitors of the cell cycle have been considered for anticancer chemotherapy. Recent studies have shown that the G_1_ phase of the cell cycle is an important period where various signals interact to determine the proliferation, quiescence, differentiation or apoptosis of cells (Sherr, 1994, 1996). Cyclins A, B, D, and E undergo periodic synthesis and degradation, thereby providing a mechanism to regulate cyclin-dependent kinase (CDK) activity throughout the cell cycle (King and Cidiowski, 1998). D- and E-type cyclin are expressed during G_0_/G_1_ and are referred to as start cyclins. A CDK can only be activated once its partner cyclin has reached a critical concentration (King and Cidiowski, 1998). The CDK2-cyclin E complex, cyclin D-dependent kinases 4 and 6, and phosphorylated Rb are necessary for transition from G_1_ to S phase (Tsai *et al*, 1993; Ohtsubo *et al*, 1994).

The use of chemical agents to induce differentiation of tumour cells has received widespread attention as a potentially less toxic cancer therapy. Human colon cancer cells exhibit different degrees of histologic differentiation. The morphology of HT-29 cells can be modulated to express distinct differentiation markers following treatment with various inducers (Fantini *et al*, 1990; Cohen *et al*, 1999). The induction of differentiation in human colon cancer cells is associated with an upregulation of production of the differentiation-related molecules fibronectin (FN) and carcinoembryonic antigen (CEA) (Drewinko *et al*, 1976; Brattain *et al*, 1984; Huang and Chakrabarty, 1994).

New chemotherapeutic agents such as topoisomerase I inhibitor irinotecan (CPT-11) are now used as a second-line chemotherapeutic agent for patients who have failed to respond to previous 5-FU-based chemotherapy, but the survival remains poor for patients with metastatic colorectal carcinoma (Cao and Rustum, 2000). In this study, we evaluated the *in vitro* effects of DPD on proliferation and induction of differentiation of colon cancer cells. In addition, we examined the *in vivo* activity of DPD in human colon cancer cells Colo 205 xenografts. We have found that DPD produced G_1_ arrest as a result of the inhibition of the G_1_ cyclin D. In addition, DPD triggered polarisation in HT-29 cells and induced increased expression of CEA and FN production in Colo 205, HT-29 and HCT-15 cells. The *in vivo* tumorigenicity of DPD-treated cells was significantly decreased in human colon Colo 205 xenografts. Moreover, DPD was demonstrated to have *in vivo* antiproliferative effects on human colon cancer xenografts with no obvious acute toxicity.

## MATERIALS AND METHODS

### Cell culture and DPD treatment

Three colon cancer cell lines Colo 205 (ATCC: CCL-222), HT-29 (ATCC:HTB-38), and HCT-15 (ATCC: CCL-225) were used in this study. Colo 205 cells were cultured in RPMI-1640 with 10% foetal bovine serum (Hyclone, Logan, UT, USA). HT-29 cells were cultured in McCoys 5A with 10% foetal bovine serum and 0.01 mg ml^−1^ gentamycin (GIBCO, Grand Island, NY, USA). HCT-15 cells were cultured in RPMI-1640 with 20% foetal bovine serum and 0.01 mg ml^−1^ gentamycin. Cells were incubated in a humidified atmosphere of 5% CO_2_ in air at 37°C. DPD was dissolved in DMSO at a stock concentration of 10 mM and added to culture media at a final concentration of 0.5–4 *μ*M. Cells were seeded at 6 × 10^5^ cells per 60 mm or 1 × 10^6^ cells per 100 mm dish in growth medium. The following day the cells were replenished with medium containing the DPD. Cells were harvested and counted by haemocytometer at 24, 48, and 72 h after treatment with DPD and used for further analysis.

### Assessment of cell viability

At appropriate times after DPD exposure, attached cells were trypsinised and combined with nonadherent cells. After centrifugation, cells were resuspended in culture media and stained with 0.4% trypan blue, and viable cells were counted using a haemocytometer.

### DNA staining

Cycle TEST™ PLUS DNA Reagent Kit (Becton Dickinson, San Jose, CA, USA) was used for DNA staining. After washing the cells twice with buffer solution, the cell concentration was adjusted to 1.0 × 10^6^ ml^−1^ and 0.5 ml of cell suspension was centrifuged at 400 **g** for 5 min at room temperature (20–25°C). The cell pellet was added on 250 *μ*l of solution A (trypsin buffer) and gently mixed. After incubation at room temperature for 10 min, 200 *μ*l of solution B (trypsin inhibitor and RNase buffer) was added to each tube, gently mixed and then incubated at room temperature for 10 min. This was followed by with the addition of 200 *μ*l of solution C (propidium iodide (PI) stain solution) and incubated for 10 min in the dark on ice (2–8°C). The sample was filtered through a 50-mm nylon mesh and used for flowcytometric analysis.

### Cyclin D staining

Cells were washed with 5 ml of wash buffer (PBS (0.1%), NaN_3_ (1%), heat-inactivated foetal bovine serum), and fixed in 5 ml of 1% methanol-free formaldehyde in PBS for 15 min on ice. Cells were then centrifuged at 400 **g** for 5 min and the supernatant was discarded. Cold 75% ethanol was added drop by drop to the cell pellet and incubated at −20°C for a minimum of 2 h. Just prior to staining, ethanol was removed by centrifugation at 400 **g** for 10 min. Cold 0.25% Triton X-100 5 ml wash buffer was added to the cell pellet, vortexed and incubated for 5 min on ice. A measure of 20 *μ*l of FITC-conjugated mouse anti-human cyclin D_1_, D_2_, D_3_ monoclonal antibody (clone G124-259, BD PharMingen) (Darzynkiewicz *et al*, 1996) or isotype control antibody (mouse IgG_1_, clone MOPC-21) was added to 100 *μ*l cell suspension (1 × 10^6^ cells) and incubated for 30 min at room temperature in the dark. The cells were washed with wash buffer, and then stained with PI solution (10 *μ*g ml^−1^) for 10 min. The sample was filtered through a 50-mm nylon mesh and used for flow cytometric analysis.

### Flow cytometry

Cells (20 000) were analysed on a FACSCalibur flow cytometer (Becton Dickinson) using an argon-ion laser (15 mW) with the incident beam at 488 nm. The red fluorescence (PI) was collected through a 585-nm filter and the green fluorescence (fluorescein isothiocyanate) was collected through a 530-nm filter. The data were analysed using ModFit and Cellquest softwares on Macintosh computer.

### FN and CEA production

Cells (1 × 10^6^ per dish) were seeded on 10-cm dishes and allowed to attach overnight, and then the medium was discarded and replenished with medium containing DPD for incubation at 37°C for 3 days. The conditioned medium was collected and stored at −20°C before analysis. The levels of FN production were measured by a quantitative enzyme-linked immunosorbent assay (ELISA, Chemicon, USA), using primary rabbit anti-human FN antibody and goat anti-rabbit peroxidase conjugated secondary antibody. The levels of CEA production were measured by a radioimmunoassay (RIA) kit (CIS Bio International, France). Both FN and CEA concentration were normalised to nanograms per 1 × 10^6^ cells. The results are expressed as an average of duplicate assays from one of two independent experiments.

### Analysis of the reversibility of DPD-induced growth inhibition of Colo 205 cells

Cells (1 × 10^6^ per dish) were seeded on 10-cm dishes and allowed to attach overnight and then the medium was discarded and replenished with medium containing the DPD for incubation at 37°C for 3 days. At the end of 3 days, the medium was discarded, and the cells were replenished with fresh medium. Cells were harvested and the cells viability were examined by haemocytometer at days 0, 3, 4 and 5 after withdrawal from 1, 2, or 4 *μ*M DPD treatment for 72 h.

### Scanning electron microscopy (SEM)

The method of Cohen *et al* (1999) was used. Briefly, cells (1 × 10^6^ per dish) were seeded in 10-cm dishes and allowed to attach overnight, and then the medium was discarded and replenished with medium containing DPD for incubation at 37°C for 3 days. At the end of 3 days the DPD was withdrawn, the cells were replenished with fresh medium. At 1 week after DPD withdrawal, cells were washed with PBS, then fixed with 2% glutaraldehyde in PBS then post-fixed with 1% OsO_4_ in PBS, dehydrated in ethanol, dried, coated with gold and examined in a field emission SEM (JSM-6500F, Jeol, Japan).

### *In vivo* analysis of the tumorigenicity of DPD-treated Colo 205 cells

All the *in vivo* experiments have been carried out with ethical committee approval, and meet the standards required by the UKCCCR Guidelines (Workman *et al*, 1998). The 8-week-old male BALB/c-nu nude mice were obtained from the National Laboratory Animal Center of National Applied Research Laboratories (Taipei, Taiwan), and housed in a laminar flow room under sterilised conditions with temperature maintained at 25°C and light controlled at 12 h light and 12 h dark cycle. The Colo 205 cells after 1, 2, or 4 *μ*M DPD treatment for 72 h were examined for viability and transplanted subcutaneously on both side of flank regions of nude mice. The tumour size was measured using a vernier caliper twice a week. Tumour volume (*V*) was calculated according to the following formula: *V* (mm^3^)=0.4*AB*^2^, where *A* and *B* are the longest diameter and the shortest diameter, respectively (Attia and Weiss, 1966).

### Antitumour activity of DPD in the BALB/c-nu nude mice tumour xenograft model

Colo 205 cells were harvested and resuspended in serum-free RPMI-1640 medium. Cells were adjusted to 1 × 10^7^ cells ml^−1^, and inoculated in nude mice as described above. Each experimental group included five to six mice bearing bilateral tumours. DPD was dissolved in DMSO, treatment started when tumour size was 3–5 mm. DPD was administered via i.p. injection once a week at doses of 18.8, 37.5 or 75 mg kg^−1^ (volume of injection: 0.1 ml per 20 g of body weight), respectively. The control group received DMSO vehicle. Tumour size and body weight were monitored twice a week throughout the experiment. Tumour size was measured as described above. Drug efficacy was assessed as tumour growth index (TGI)=*V*_*n*_/*V*_0_, where *V*_*n*_ is the tumour volume of treated group on day *n* and *V*_0_ is the initial tumour volume. At day 32, all mice were killed by CO_2_ gas. Tumours, livers, kidneys, and lungs were collected, fixed, embedded, and stained with haematoxylin and eosin for pathological analysis.

### Enhancement of the antitumoral activity of chemotherapeutic agent CPT-11 by DPD

Colo 205 cells were treated with DPD (4 *μ*M) or vehicle (DMSO) for 24 h. Then the cells were further treated with or without CPT-11 (25 *μ*g ml^−1^) for another 72 h. The antitumoral activities were assayed by MTT method. The detailed procedure was reported in a previous study (Wang *et al*, 1997). The cell cycle progression was analysed as described above.

### Statistics

All data are expressed as mean±s.e. The difference between groups was assessed using Student's *t*-test. A *P*<0.05 is considered as significant difference.

## RESULTS

### Antiproliferative effects of DPD

**Figure 1:**
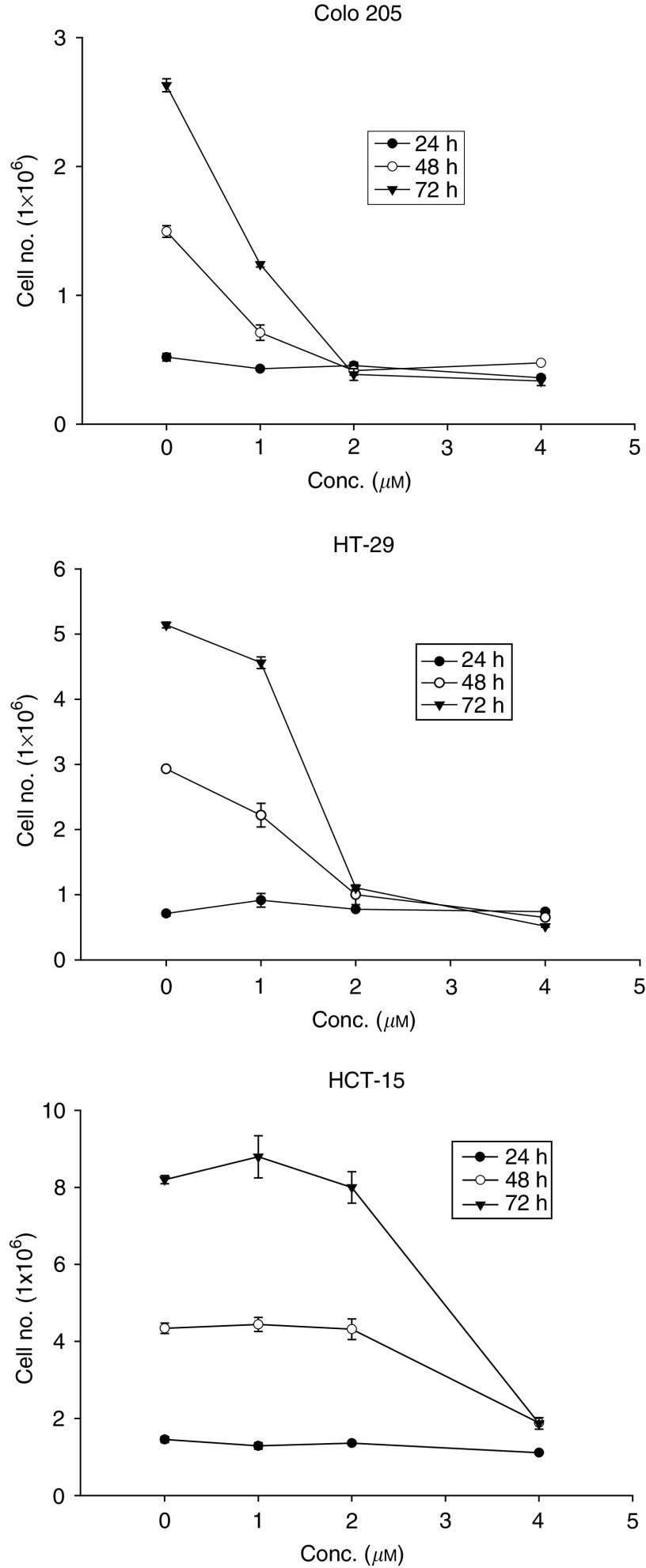
Effect of DPD on the growth of three human colon cancer cell lines. Cells were seeded at 5 × 10^5^ cells per 60 mm or 1 × 10^6^ cells per 100 mm dish in growth medium. The following day the cells were replenished with medium containing 1, 2, or 4 *μ*M DPD. Cells were harvested and counted by haemocytometer. Each point represents the mean±s.e. of triplicate cultures.

To determine the ability of DPD to inhibit cell proliferation in three colon cancer cell lines, cells were incubated in the absence or presence of increasing concentrations of DPD for 1–3 days. As shown in Figure 1

, we observed an inhibition of cell growth in DPD-treated cells as compared with vehicle controls. Significant growth inhibition was observed at days 2 and 3 in Colo 205 cells and HT-29 cells after treatment with 1, 2, and 4 μM DPD, respectively. In the relatively resistant cell line HCT-15, growth inhibition was observed after treatment with 4 μM DPD at days 2 and 3.

### Cell cycle analysis

**Figure 2:**
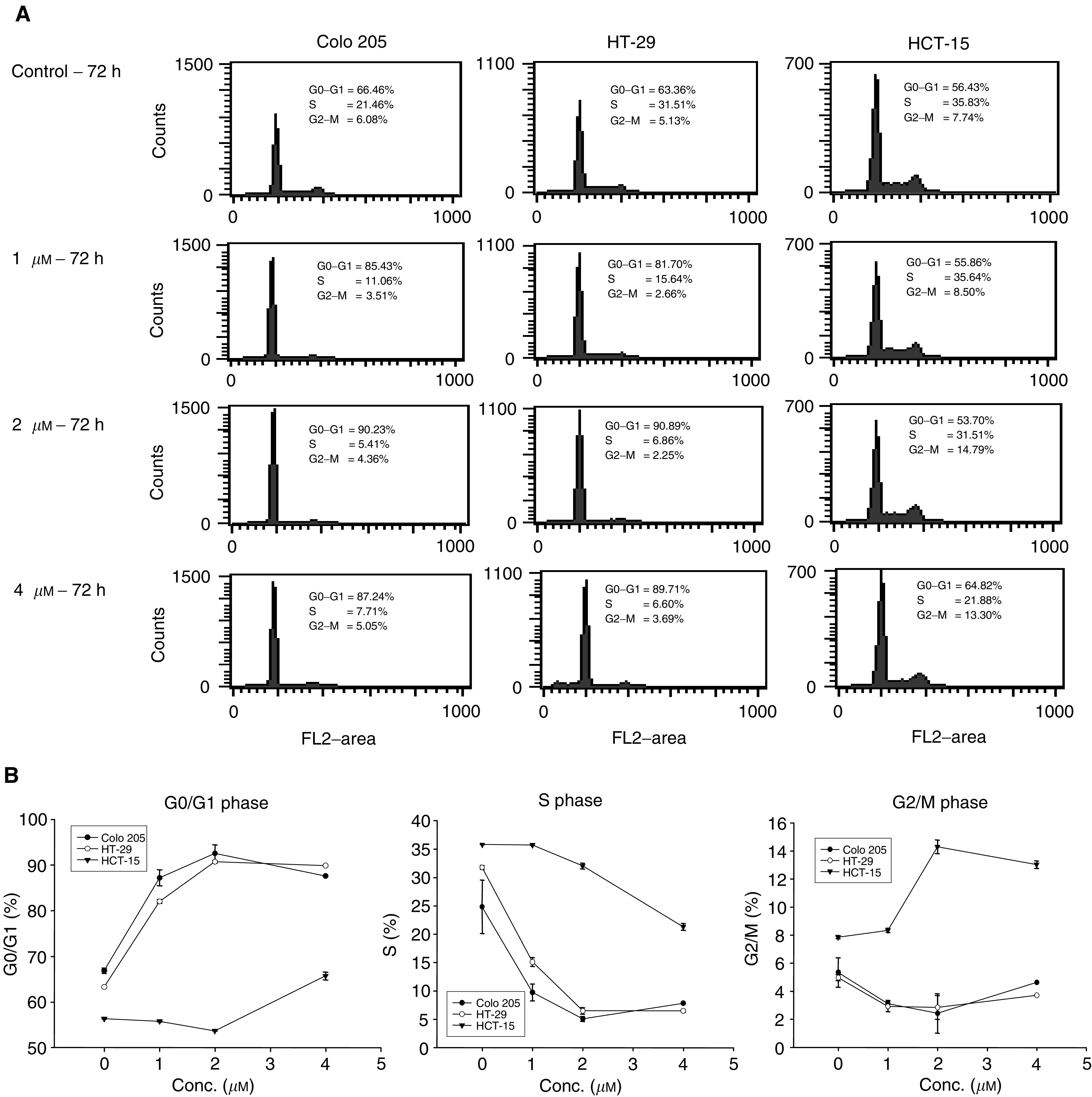
The effect of DPD on cell cycle arrest at day 3 in three colon cancer cell lines. Cells were seeded at 5 × 10^5^ cells per 60 mm or 1 × 10^6^ cells per 100 mm dish in growth medium. At the following day the cells were replenished with medium containing 1, 2, or 4 *μ*M DPD. Cells were harvested and the cell cycle was analysed by flow cytometry as described in ‘Materials and Methods’. (**A**) The representative cell cycle progression in DPD-treated colon cancer cell lines. (**B**) The mean percentages of G_0_/G_1_, S and G_2_/M cells in DPD-treated colon cancer cell lines. Data are shown as the mean±s.e. of mean of duplicate samples from one of two independent experiments.

The cell cycle progression of Colo205, HT-29 and HCT-15 cells was examined using flow cytometry after exposure to 1, 2, or 4 μM DPD for 72 h (Figure 2A

). Figure 2B shows that majority of Colo 205 cells accumulated in G_1_ phase (87.2%) with a decrease of cells in S phase (9.7%) after treatment with 1 μM DPD for 72 h. Colo205 and HT-29 cells were mainly in G_0_/G_1_ phase (88–93%), and only a few percent of cells in S phase (5–8%) and the G_2_/M phase (2.3–5%) after exposure to 2 or 4 μM DPD for 72 h. The moderately increased G_0_/G_1_, G_2_/M phase and decreased S phase of HCT-15 cell populations were observed after their exposure to 2 or 4 μM DPD for 72 h. These results showed that treatment of colon cancer cells with DPD resulted in increased G_0_/G_1_ phase with concomitant decrease of cells in S phase.

### Expression level of cyclin D in DPD-treated Colo 205 and HT-29 cells

**Figure 3:**
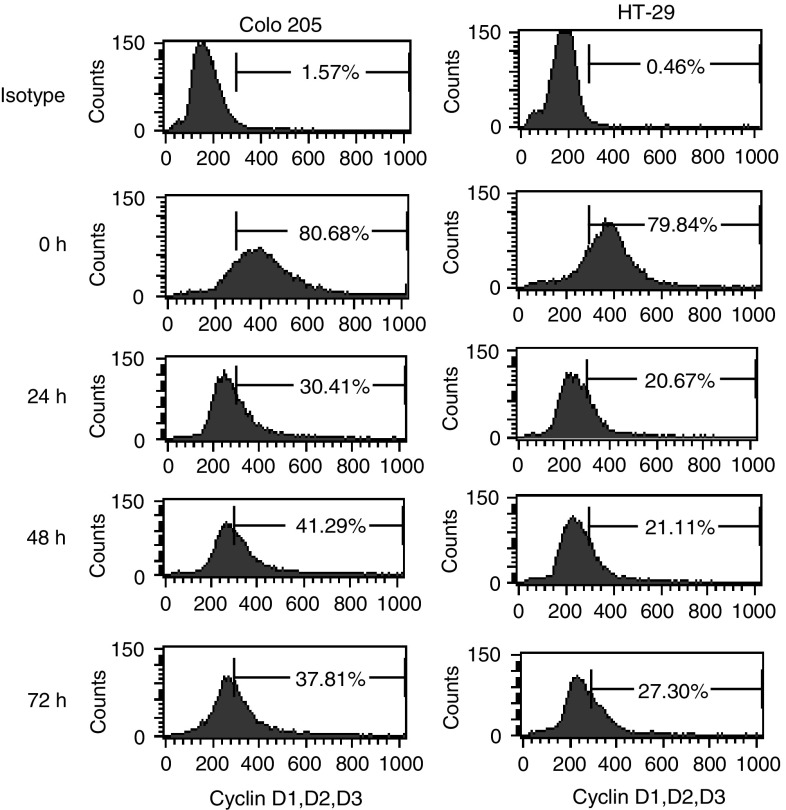
Expression level of cyclin D protein was analysed by flow cytometry after treatment with 2 *μ*M DPD for 0–72 h. The living (PI negative) cells were collected and values indicate the percentage of cells with cyclin D positive.

To further confirm the cytostatic effect induced by DPD, the expression of cyclin D was analysed by flow cytometry. After 2 *μ*M DPD treatment for 0–72 h, the percentage of cells with expression of cyclin D in Colo 205 and HT-29 cells decreased from 80.68 to 26.46% and 79.84 to 24.49%, respectively (Figure 3

).

### Induction of CEA and FN expression

Figure 4

**Figure 4:**
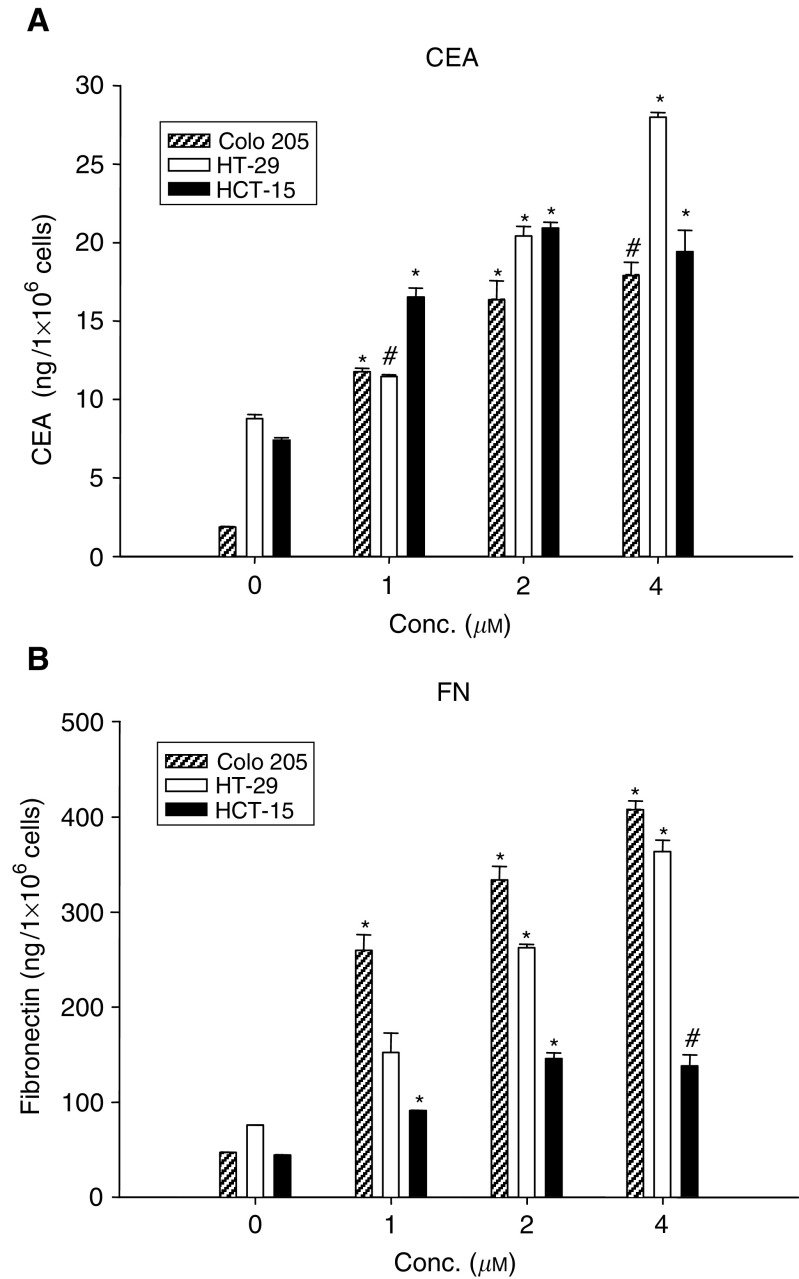
Induction of CEA (**A**) and FN (**B**) production by DPD. Conditioned medium from cells treated with 1, 2, or 4 *μ*M DPD for 72 h was analysed for CEA and FN production by assay kit. CEA and FN concentrations were normalised to ng ml^−1^ 1 × 10^6^ cells. Data are shown as the mean±s.e. of the mean of duplicate assays from one of two independent experiments. ^*^P<0.01; # *P*<0.05, compared to the respective control group.

illustrates the induction of CEA and FN production in Colo 205, HT-29, and HCT-15 cells after DPD treatment for 72 h. DPD treatment of these cells resulted in increased secretion of CEA (Figure 4A) and FN (Figure 4B) into the culture medium. *A* greater than 10-fold increase in soluble CEA and eight-fold increase in soluble FN/10^6^ DPD-treated Colo 205 cells was seen compared to the untreated control cells. The HT-29 and HCT-15 cells also showed a more than three-fold increase in CEA and FN production after treatment with DPD.

### Irreversible effect of DPD-induced growth inhibition of Colo 205

**Figure 5:**
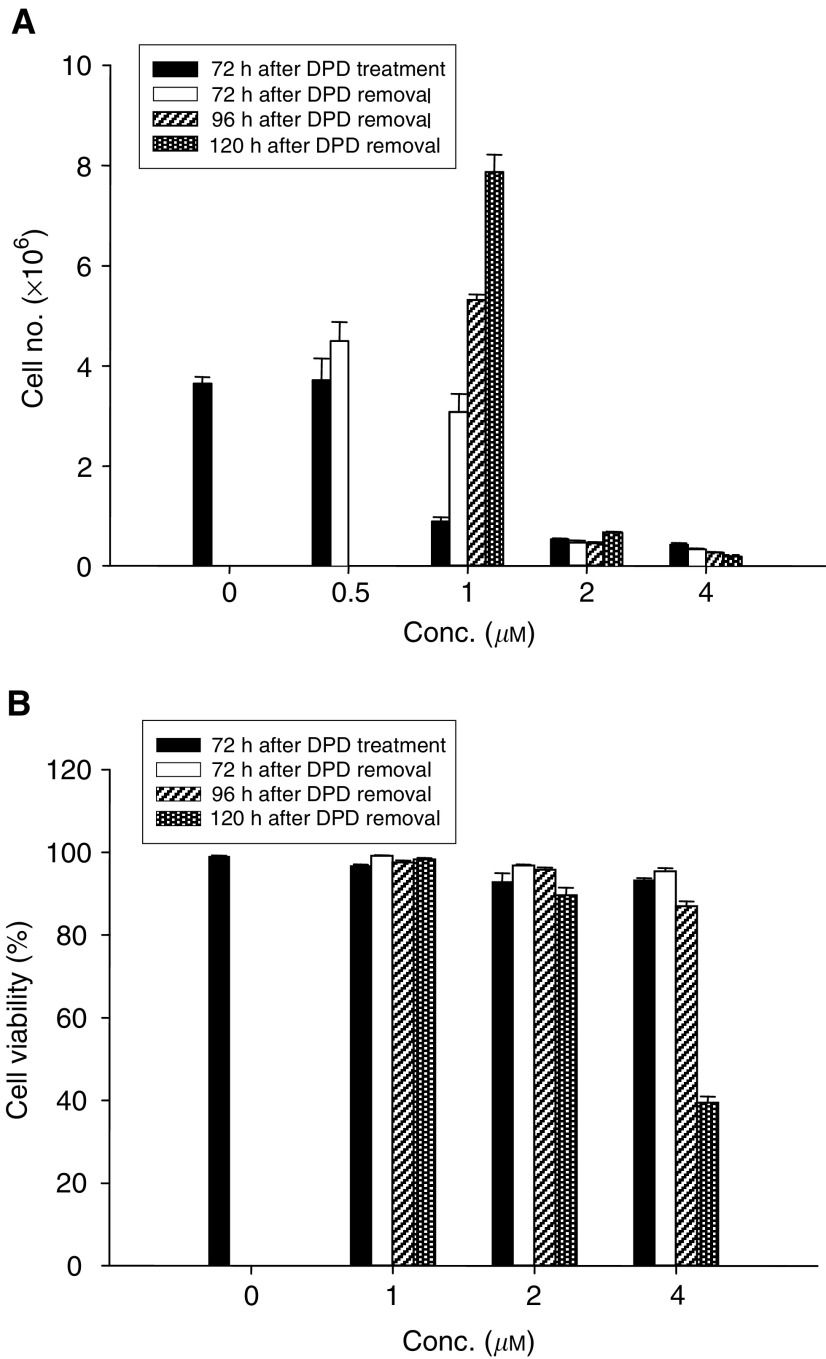
(**A**) Irreversible effect of DPD-induced growth inhibition of Colo 205. Cells were seeded at 1 × 10^6^ cells per 100 mm dish in growth medium. The following day the cells were replenished with medium containing 0.5, 1, 2, or 4 *μ*M DPD. Cells were harvested at days 0, 3, 4, 5 after 1, 2 or 4 *μ*M DPD treatment for 72 h, then withdrawn. (**B**) The cell's viability was examined by haemocytometer. Each point represents the mean±s.e of triplicate cultures.

To further investigate whether the DPD-induced growth inhibition was reversible, Colo 205 cells were treated with DPD for 72 h, and the cells were withdrawn from DPD by culturing in fresh medium for another 120 h. As shown in Figure 5A

, at 72–120 h after removal of 1 *μ*M DPD from the medium the proliferation activity of Colo 205 was reversed and returned to control level. However, no reversal effect was observed after 2 or 4 *μ*M DPD treatment. The viabilities of treated or nontreated cells were 90–95%, except the cells at 120 h after 4 *μ*M DPD treatment (40%) (Figure 5B).

### Effect of DPD on brush border formation

**Figure 6:**
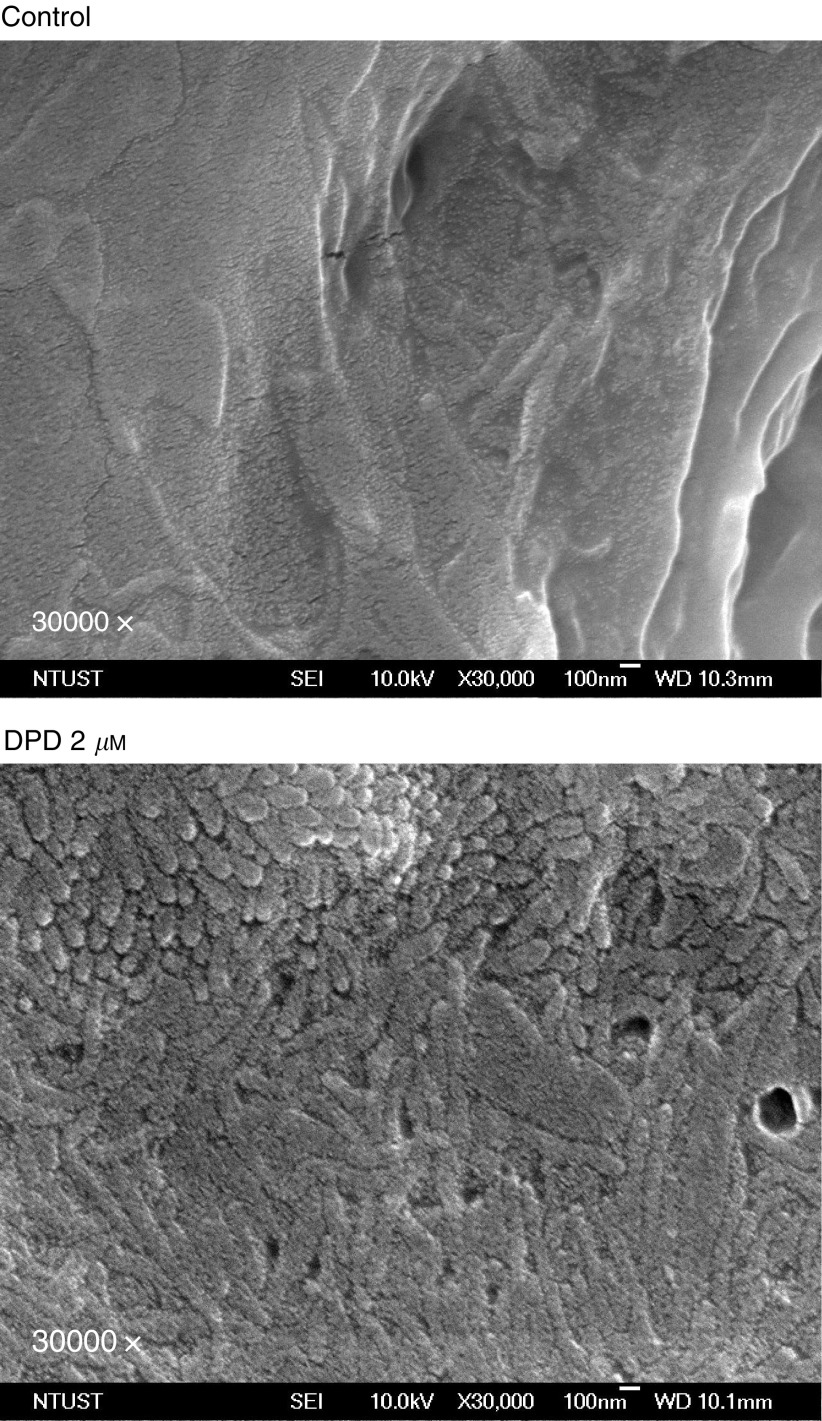
Scanning electron micrographs of HT-29 cells. Cells were treated with 0.1% DMSO (control) or 2 *μ*M DPD for 72 h, then DPD withdrawn and replenished with fresh medium. The morphology of cells was examined 1 week after DPD withdrawal.

To further investigate whether DPD-induced epithelium like brush border formation in HT-29 cells. Cells were treated with 2 *μ*M DPD for 72 h, then cells were replenished with fresh culture medium and cultured for another week. Figure 6

shows that prominent brush border formation was observed in 2 *μ*M DPD-treated cells but not in control cells.

### *In vivo* tumorigenicity of DPD-treated Colo 205 cells

**Figure 7:**
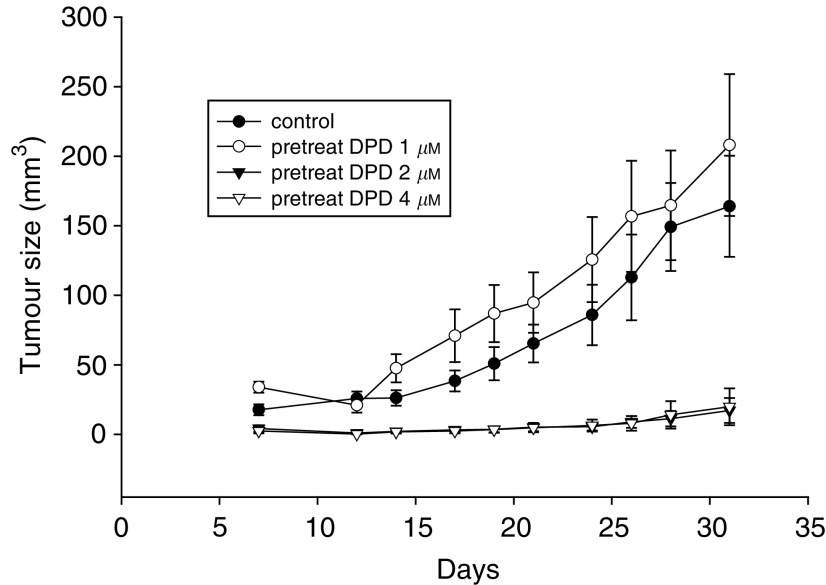
*In vivo* tumorigenicity of DPD-treated Colo 205 cells. To evaluate the *in vivo* antiproliferative effect of colo205 cells after 1, 2, or 4 *μ*M DPD treatment for 72 h, then withdrawal, the cell's viability was examined and transplanted into BALB/c nude mice and the tumour size was measured twice a week. The viability of treated Colo 205 was greater than 95%. The tumour incidences were 10 out of 10 (control), nine out of 10 (1 *μ*M), four out of 10 (2 *μ*M), and two out of 10 (4 *μ*M), respectively. Each data point is the mean±s.e. from 10 samples of one representative experiment. The tumorigenicity of 2 or 4 *μ*M DPD-treated Colo 205 was significantly (*P*<0.01) reduced.

To evaluate whether DPD-treated cells are still tumorigenic, Colo 205 cells were treated with 0, 1, 2, or 4 *μ*M DPD for 72 h (cell viability: 98.3, 98.8, 97.5, and 97.0%, respectively), and then transplanted into BALB/c nude mice. Figure 7

shows that tumours from control animals grew to an average size of 164 mm^3^ at day 32. The tumour of DPD (1 *μ*M)-pretreated Colo 205-implanted animals was marginally larger than control. However, tumours from 2 to 4 *μ*M DPD-pretreated Colo 205 implanted animals grew to an average size of only 17–20 mm^3^. The tumour incidences were 10 out of 10 (vehicle control), nine out of 10 (1 *μ*M), four out of 10 (2 *μ*M) and two out of 10 (4 *μ*M), respectively. These results showed that the tumorigenicity of 2–4 *μ*M DPD-treated Colo 205 cells was significantly (*P*<0.01) reduced.

### *In vivo* antiproliferation effect of DPD for human colon cancer xenografts

**Figure 8:**
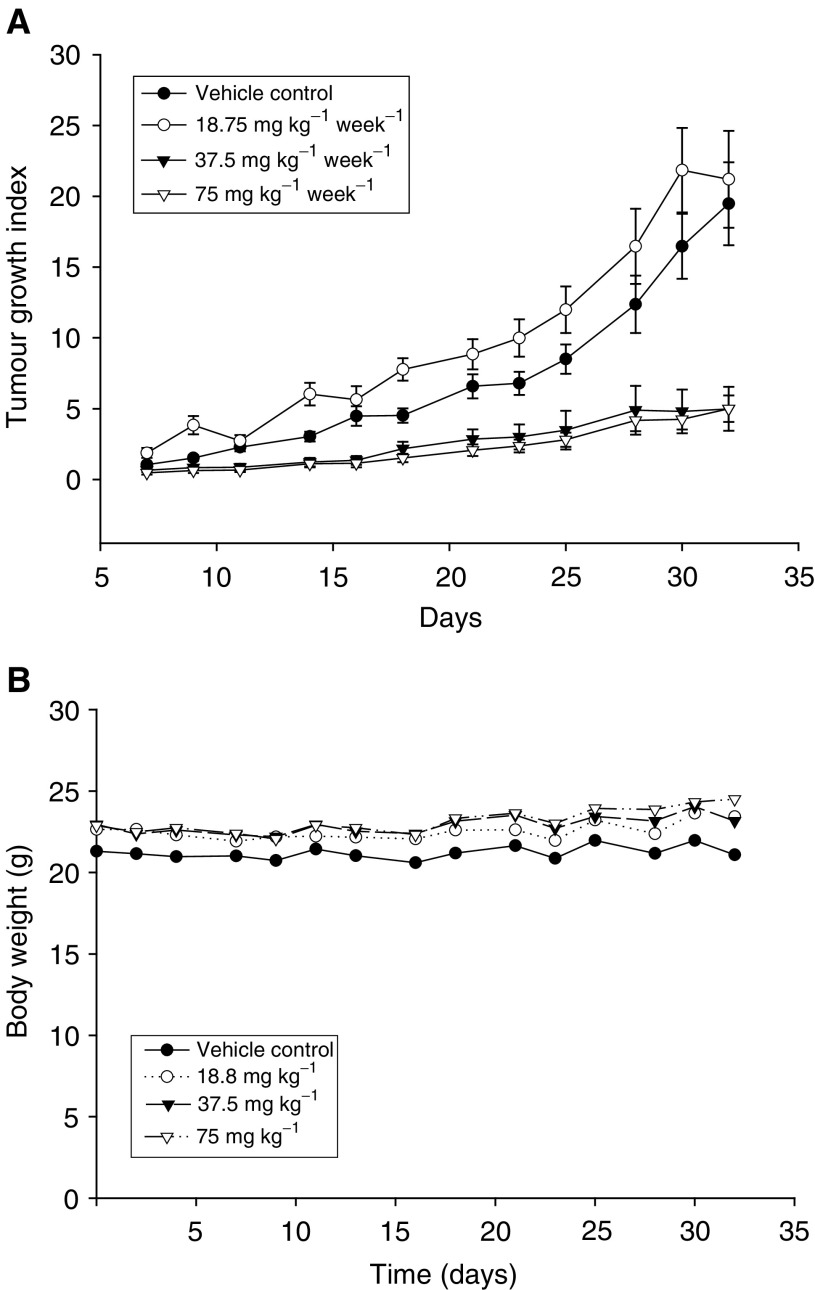
(**A**) *In vivo* antiproliferaive effect of DPD for human colon cancer xenografts. When the tumours were palpable (3–5 mm), the BALB/c nude mice were either treated with vehicle control or DPD (i.p., once a week). Each data are the mean±s.e. from six to 10 samples of one representative experiment. Treatment of nude mice with DPD (37.5–75 mg kg^−1^), the tumour growth index was significantly (*P*<0.05) decreased in mice as compared to control groups. (**B**) Changes in body weight of nude mice after treatment with DPD. Each data point is the mean±s.e. from six to 10 samples of one representative experiment.

We further examined whether DPD is also effective *in vivo* after tumour formation. Cancer cells were transplanted into BALB/c nude mice, and when the tumours were palpable (3–5 mm), the mice were treated either with vehicle control or DPD (18.75–75 mg kg^−1^, i.p., once a week). Treatment of nude mice with DPD (37.5–75 mg kg^−1^), the tumour growth index was significantly (*P*<0.05) decreased in mice as compared to control groups at the end of experiment. Figure 8A

shows that tumour growth index from control animals showed an average of 19.48 at the end of this study. In contrast, the tumour growth index from DPD (37.5 or 75 mg kg^−1^)-treated animals had an average of only 4.99. The tumour growth index of DPD (18.75 mg kg^−1^)-treated animals was slightly larger than control but not statistically significant.

The challenge of DPD (18.75–75 mg kg^−1^, i.p., once a week) in nude mice produced no obviously acute toxicity. No significant reduction in body weight was found in DPD-treated mice (Figure 8B). In addition, no tissue damage was observed in the liver, lung and kidney after examination of the tissue slices stained with haematoxylin and eosin (data not shown).

### Enhancement of the antitumoral activity of chemotherapeutic agent CPT-11 by DPD

**Figure 9:**
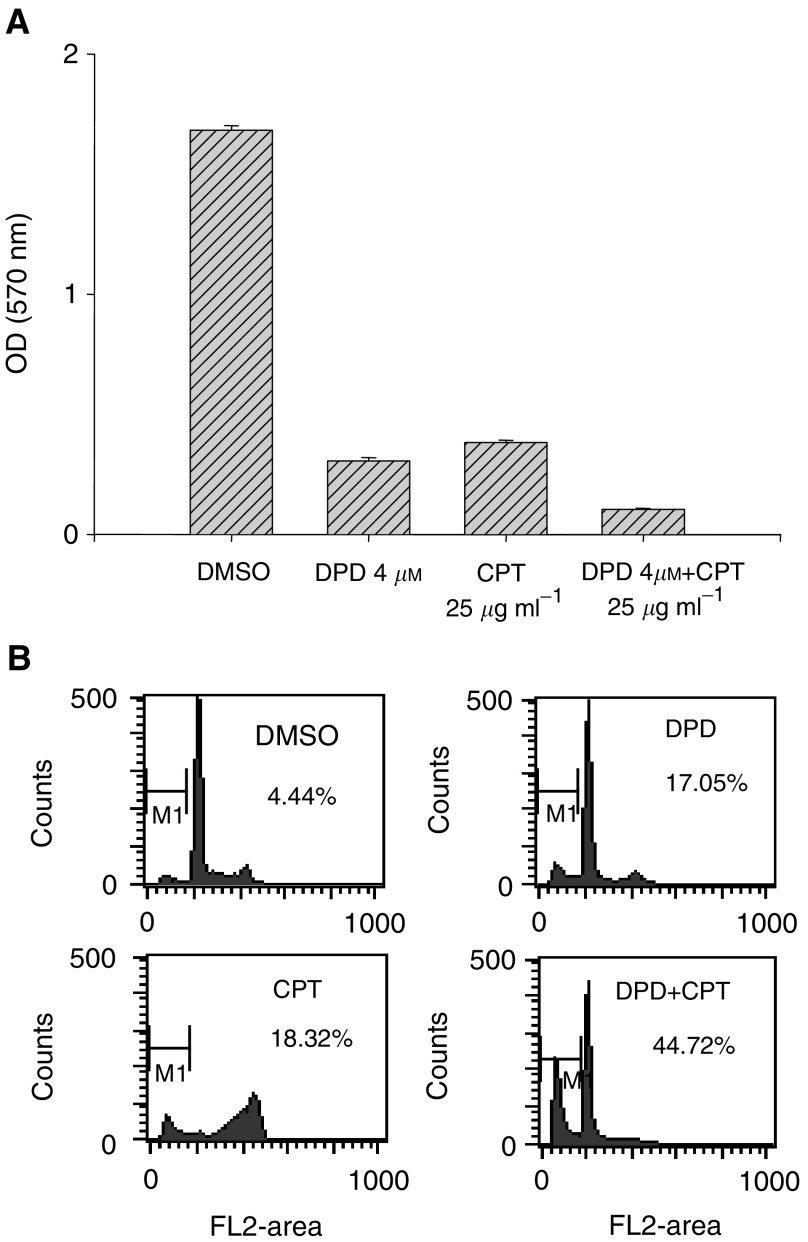
Enhancement of the antitumoral activity of CPT-11 by DPD. Colo 205 cells were treated with DPD (4 *μ*M) or vehicle (DMSO) for 24 h. Then the cells were further treated with or without CPT-11 (25 *μ*g ml^−1^) for another 72 h. (**A**) The antitumoral activities of each treatment were assayed by MTT method. Each data point is the mean±s.e. from six samples of one representative experiment. (**B**) The representative cell cycle progressions in treated cells are from one of the two independent experiments.

To further investigate whether DPD could enhance the antitumoral activity of the chemotherapeutic agent CPT-11. Colo 205 cells were treated with DPD or DPD in combination with CPT-11 and their cell cycle analysed. As shown in Figure 9A

, the antitumoral activity of DPD combined with CPT-11 showed a three-fold increase in treated cells as compared to DPD or CPT-11 alone. Figure 9B shows that the population of sub-G_0_/G_1_ cells of DPD combined with CPT-11 treatment group was increased (44.7%) as compared to DPD (17.1%) or CPT-11 (18.3%) alone. These results clearly showed that DPD enhanced the antitumoral activity of the chemotherapeutic agent CPT-11.

## DISCUSSION

We have previously found that diamantane derivatives exert strong growth inhibitory activities *in vitro* against several cancer cell lines in National Cancer Institute (NCI) Anticancer Drug Screen. Therefore, in the current study, we evaluated the effects of DPD *in vitro* and *in vivo* on the human colon cancer cells. To the best of our knowledge, the present study is the first report that refers to the *in vitro* cytostatic and differentiation promoting and *in vivo* antiproliferative effectiveness of DPD.

In the current study, three colon cancer cell lines with distinct biological properties (i.e. morphological differentiation, CEA production, etc.) were used and treated with DPD. The Colo 205 is a poorly differentiated cell line, and HT-29 is a well differentiated cell line (Kondoh *et al*, 1992; Laferte and Loh, 1992). The two colon cancer cell lines regardless of their state of differentiation had their growth markedly slowed by DPD. However, the multidrug-resistant HCT-15 (Stein *et al*, 1996) also showed response to DPD. The cytostatic potency of DPD in three cancer cell lines was heterogenous. Colo 205 and HT-29 were more sensitive than HCT-15 in DPD-induced cell growth suppressing and concomitant cell cycle arrest. The multidrug-resistant HCT-15 also showed different patterns of cell cycle histogram from the Colo 205 and HT-29. In eukaryotes, the cell cycle is tightly regulated by several protein kinases composed of a CDK subunit and corresponding regulatory cyclin subunit, and CDK inhibitors (Grana and Reddy, 1995; Lees, 1995). Cyclin E is a driving force in the G_0_/G_1_-to-S phase transition in the cell cycle, and the cyclin D is a start cyclin in cell cycle. The decrease of cyclin D in specific cell types may signal a switch between proliferation and differentiation (Gillett and Barnes, 1998). In the present study, we have demonstrated that within 72 h of exposure, DPD could mediate accumulation of Colo205 and HT-29 cells in G_1_ phase (>90%), and the expression of cyclin D was dramatically decreased in DPD treated cells. The data suggest that the presence of DPD is promoting the loss of cyclin D from the cell in response to some as yet undefined process involved in DPD-induced G_0_/G_1_ arrest and differentiation.

Differentiation-inducing chemicals often inhibit growth in conjunction with the induction of differentiation in cancer cells. In many transformed cell types, the induction of differentiation is associated with an increase of FN expression (Reynolds *et al*, 1998). All-*trans* retinoic acid (ATRA) and sodium butyrate (NaB) have also been shown to upregulate FN production in human colon cancer HT-29 cells (Ohannesian *et al*, 1994). This study shows that the FN production was increased by DPD in three cell lines. This result was similar to ATRA and NaB in HT-29 cells. The Colo 205 cells, however, were relatively responsive to DPD in FN production by comparison with the HT-29 and HCT-15. DPD induced a three-fold increase in FN production in the HT-29 cells, which was similar to ATRA (Niles *et al*, 1988).

Several studies have shown that the upregulation of CEA expression is also associated with a differentiation induction response in human colon cancer cells (Tanaka *et al*, 1990; Schroder *et al*, 1998). CEA is an intracellular adhesion glycoprotein (Toribara *et al*, 1989). ATRA and NaB have been reported to enhance CEA production in some colon cancer lines (Velcich *et al*, 1995; Wang *et al*, 1999). DPD was a potent inducer of soluble CEA production in the three cell lines, which produce CEA constitutively. These results are in agreement with observations that NaB and ATRA induced differentiation in colon cancer cells (Wang *et al*, 1999). Nevertheless, NaB, at millimolar dose, induced colon cancer cells to differentiate. However, the NaB-mediated HT29 differentiation was found to be reversible following a return to NaB-free medium (Ophir *et al*, 1995). *In vivo*, NaB has a half-life too short to produce any therapeutic effect. Our results show that DPD is more active than NaB in suppressing cell growth and concomitantly promoting differentiation of HT-29 colon cancer cells. Differential cellular responses were observed in these three cell lines after treatment with DPD. The differences in the cellular responses to DPD may be due to the differences in the biological properties of the three cell lines.

The human colon cancer cell line HT-29 offers a favourable study system for the evaluation of various inducers involved in differentiation (Fantini *et al*, 1990). Differentiation was characterised by flattening of the cells, formation of brush borders (epithelium-like structure) on apical HT-29 cell surface and tight junctions between adjacent cells. All these morphological observations clearly indicate that agents known to arrest cell cycle are involved in the induction of differentiation and apoptosis in HT-29 cells (Fantini *et al*, 1990). In this study, DPD-induced Colo 205 and HT-29 cell cycle arrest. These cells differentiate to become bigger in size, less round and express island shape, and increased adherence to one another. The DPD-induced morphology changes are similar to forskolin in HT-29 cells (Fantini *et al*, 1990), but these cells were cultured for a long-term period (21 days) and then treated with forskolin. However, no reversal in these morphological changes was found after withdrawal of DPD in Colo 205 and HT-29 cells even after 120 h. The brush borders on HT-29 cell surface were also demonstrated by SEM at 1 week after DPD treatment and withdrawal. These results highlight the important properties of irreversible differentiation effect of DPD. One possibility is that the relatively lipophilic nature of DPD reduces cellular efflux upon rinsing of drug-containing medium after the initial drug exposure. The continued growth inhibitory activity may be due to residual intracellular drug.

Treatment of Colo 205 tumour-bearing nude mice with DPD significantly decreased the tumour growth index in mice as compared to control groups. Moreover, once a week i.p. challenge of DPD in nude mice produced no obvious acute toxicity. These results together with the data that combination therapy showed DPD could enhance the antitumoral activity of the chemotherapy agent CPT-11 in colon cancer Colo 205 cells suggest that a G1-targeting and differentiation-inducing agent DPD, has potential for combination with other antineoplastic agents in treatment of human colon cancer cells.
